# Bioinformatic Analysis of microRNAs Associated with Chemotherapy-Induced Cognitive Impairment: Integration of Gene Networks and Neuroinflammatory Pathways

**DOI:** 10.3390/biomedicines14030594

**Published:** 2026-03-06

**Authors:** Lucas Volpi Cândido, Marcos Otávio Bueno, Ricardo Cervini, Natan Veiga, Claudriana Locatelli, João Paulo Assolini, Gustavo Colombo Dal-Pont, Ariana Centa

**Affiliations:** 1 Curso de Medicina, Universidade Alto Vale do Rio do Peixe (UNIARP), Caçador 89500-199, Brazil; 15472855675@uniarp.edu.br (L.V.C.); 11791090907@uniarp.edu.br (M.O.B.); ricardocervini@hotmail.com (R.C.); natan.veiga@uniarp.edu.br (N.V.); claudriana@uniarp.edu.br (C.L.); joao.assolini@uniarp.edu.br (J.P.A.); gustavo.colombo@uniarp.edu.br (G.C.D.-P.); 2 Laboratório de Pesquisa Translacional em Saúde, Universidade Alto Vale do Rio do Peixe (UNIARP), Caçador 89500-000, Brazil; 3 Programa de Pós-Graduação em Desenvolvimento e Sociedade, Universidade Alto Vale do Rio do Peixe (UNIARP), Caçador 89500-199, Brazil

**Keywords:** chemobrain, micrornas, neurotoxicity, chemotherapy, neuroinflammation

## Abstract

**Background/Objectives**: Neuropsychological changes induced by cancer and its treatments, especially chemotherapy, represent a significant clinical challenge, being responsible for persistent cognitive deficits known as chemobrain. This study aimed to identify microRNAs (miRNAs) associated with these alterations, map their interaction networks, and determine the main biological pathways involved. **Methods**: An integrative review and in silico analysis were conducted to study the role of microRNAs. **Results**: Six experimental studies using animal models were selected, which showed that agents such as doxorubicin, cisplatin, and methotrexate induce changes in domains such as memory, attention, and learning. Among the analyzed miRNAs, miR-155-5p, miR-21-5p, and miR-125b-5p stood out, being associated with pathways related to neuroinflammation, oxidative stress, apoptosis, and synaptic dysfunction. Computational analyses revealed that these miRNAs act on pathways such as MAPK, PI3K-Akt, mTOR, neurotrophins, and cytokine receptors. The interaction analysis among target genes also revealed a functionally connected network, with coordinated involvement in inflammation, neuronal apoptosis, and glial differentiation processes, suggesting a role in cellular stress responses and neuroinflammatory pathologies. **Conclusions**: These findings suggest that miRNAs play a central role in mediating the observed neurocognitive changes and may represent promising biomarkers and therapeutic targets to mitigate the effects of chemobrain. The study also highlights the need for future research integrating molecular and behavioral analyses to achieve more precise clinical applications.

## 1. Introduction

The neuropsychological changes induced by chemoradiotherapy comprehend a vast scope of pathophysiological transformations that affect oncologic patients [1]. The increase in exposition to conventional therapies and the new target therapies have left patients more susceptible to developing chronic and irreversible adverse effects, such as cognitive dysfunction and neuropathies [2].

Chemotherapy-induced peripheral neuropathy is one of the most cited adverse effects. However, cancer therapy also affects the domain of cognition. Cognitive impairment related to chemotherapy, also known as chemobrain, is characterized by impairments in memory, learning, attention, concentration, reasoning, processing speed, executive functions and visual–spatial skills in patients with the most diverse types of tumors [3].

Chemotherapy can activate microglia and release pro-inflammatory cytokines, which disrupt neuronal function, besides inhibiting neuronal regeneration in the hippocampus and compromise energy production in neural cells, through the formation of free radicals [4].

There are several hypotheses that try to explain the cognitive changes caused by cancer treatment, such as direct neurotoxic damage from systemic therapy and radiation, as well as chronic neuroinflammation, highlighted as the central mechanism in the pathogenesis of chemobrain. Nevertheless, there are still potential biomarkers which can denote other pathophysiological ways responsible for neuropsychological changes, like some genes and plasmatic molecules: microRNAs and exosomes [5]. Other possible pathogenic mechanisms cited include damage to progenitor cells, oxidative stress, DNA damage, apoptosis, white matter abnormalities, mitochondrial dysfunction, long-term alterations in cerebral blood flow and metabolism, loss of adaptive myelination, and glial cell circuitry. Although chemotherapy is labeled the primary cause of these changes, other factors may be responsible, such as genetic and/or epigenetic dysregulation [1].

Although the pathophysiological mechanisms of these changes are not very well understood, there is discussion about the role of direct neurotoxic effects, oxidative stress, immune system dysregulation via cytokine release and vascular damage as possible modulators of this process [6].

MicroRNAs are regulators of gene expression transcribed from introns, that is, non-coding regions of DNA. Through degradation or translational repression, depending on the degree of base pairing, miRNAs prevent mRNA translation in the ribosomes, thus acting as post-transcriptional regulators of gene expression and protein synthesis [7]. In the oncological context, miRNAs may promote tumor progression by repressing tumor suppressor genes or, conversely, slow tumor development by repressing oncogenic molecules. Translational repression and mRNA degradation mediated by miRNAs are also required for cellular differentiation and apoptosis [8,9].

The reciprocal communication between tumor cells, the microenvironment, and nervous tissue reveals molecular alterations that are still poorly understood. MicroRNAs play important roles in cell proliferation, apoptosis, tumorigenesis, and active immunity. Despite understanding miRNA–mRNA interactions, such as cleavage and translational repression, the precise mechanism and true correlation of these interactions still require further explanation. The study of microRNAs and their interactions with the organism in the field of bioinformatics represents a new frontier for understanding oncological processes [10].

The aim of this research is to identify the key microRNAs associated with chemotherapy-induced neuropsychological changes, map their protein–protein interaction networks and the biological pathways involved, and determine the main drugs related to these neurocognitive alterations.

## 2. Materials and Methods

Initially, an integrative literature review was carried out in April and May 2025 to identify scientific production related to neuropsychological changes caused by chemotherapy and carcinogenesis in cancer patients over the last ten years. The databases were last consulted on 26 May 2025.

Four electronic databases were used to search for articles: PubMed (U.S. National Library of Medicine), VHL (Virtual Health Library), SCIELO (Scientific Electronic Library Online) and ScienceDirect (Elsevier). The following subject headings were used: [chemotherapy-induced] OR [chemotherapy] AND [cognitive] OR [cognitive damage] OR [cognitive impairment] OR [chemobrain] OR [behavioral alteration] OR [behavioral impairment] OR [neurologic impairment] AND [mirna] OR [microrna]. The following inclusion criteria were also developed: timeframe within the last ten years; full text available in electronic format and written in English; and compatibility with at least one of the research objectives: to address concepts or analyses of neuropsychological changes caused by chemotherapy and tumor progression in relation to microRNAs. Articles that do not address miRNAs or chemotherapy will be excluded. It is important to note that the articles found may address animal or human populations.

Due to the specific access characteristics of each selected database, the search strategies used to search for articles were adapted to each database, guided by the subject headings mentioned above and the inclusion criteria. For every database, we used the last 10 years and English language filters. We only searched for the terms on the Title/Abstract fields. Articles published before 2015, and duplicate articles were excluded.

To guarantee the viability of this study and the reliability of the samples, due to the low availability of articles on the subject, other studies were searched in the same databases, using different descriptors: mirna AND chemotherapy-induced OR chemotherapy AND cognitive OR chemobrain.

As an integrative review, we chose not to register the protocol for the review before conducting the search.

### 2.1. miRNAs Involved in Neurological Changes Induced by Chemotherapeutic Agents—An In Silico Analysis

#### 2.1.1. miRNA Identification and Verification

Based on the theoretical research carried out in the previous stage, microRNAs were selected for computational analysis and consulted in the miRBase database [11], in order to identify the mature sequence and its conservation between species.

#### 2.1.2. Target-Genes Prediction

To identify the genes possibly regulated by these miRNAs, three tools were used: TargetScan v.8.0 [12], miRDB [13], and DIANA microT-CDS [14]. A minimum threshold, target score, or aggregated pCT of 0.8 or 80 was used for selecting genes with high confidence scores. The target genes from each database were compared to each other to identify intersections of the results using Venny 2.1 software [15].

#### 2.1.3. Ortholog Verification with the Human Genome

The main target genes found were analyzed using the Rat Genome Database [16] to confirm the existence of orthologs of the genes of the species *Rattus norvegicus* with the human genome.

#### 2.1.4. Cross-Validation with Experimental Data

The DIANA TarBase data analytics platform [17] was used to identify experimentally validated target genes of the studied miRNAs, with direct relationship experiments being selected, such as Luciferase Assay, Western blot and Quantitative Reverse Transcription Polymerase Chain Reaction (qRT-PCR).

#### 2.1.5. Enrichment of Biological Pathways

Pathway enrichment analysis is a computational biological method that identifies biological functions that are more abundant in a gene cluster than would be expected by chance and ranks such functions by their relevance [18]. Using the miRNAs identified in the literature review, the DIANA-miRPath v4.0 database [19] was used to evaluate the biological pathways of interaction between miRNAs. The corrected *p*-value (p.adj) was recommended for statistical analysis [20].

#### 2.1.6. Building Network Interactions

Mapping of protein–protein interactions between miRNAs and identified target genes was performed using STRING v.11.0 database software [21] according to high validity interaction score.

## 3. Results

### 3.1. Neuropsychological Changes Caused by Chemotherapy and Carcinogenesis and Their Relationship with miRNAs

**Figure 1:**
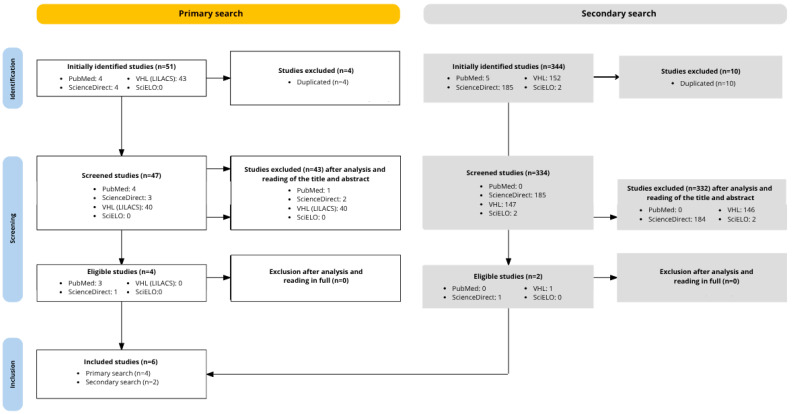
Methodological flowchart of the integrative literature review.

A total of six articles were selected for the review. The process involved two distinct sampling pools: in the first search of the 51 studies found, four were included based on the inclusion criteria. In the second search, of the 344 articles found, two studies were included after title and abstract analysis, applying the same inclusion criteria and using the open access and research articles filters to facilitate the search. Finally, 375 articles were excluded due to duplication or not addressing the research topic. In total, six studies published between 2017 and 2025 were used. A flowchart was created to illustrate the research steps (Figure 1).

All articles used are original experimental studies. All experimental studies used rodents as in vivo models. All six studies analyzed hippocampal alterations, while only a few included the prefrontal cortex and the blood–brain barrier. All studies addressed microRNAs or the microRNAome.

The included studies were gathered in Table 1, which presents their main characteristics, covering the type of study, the population analyzed, the main areas of the nervous system investigated and their main objectives.

The detailed molecular findings of each study are presented in Table 2, which summarizes information on the chemotherapeutic agents involved, as well as the experimental conditions. The table also highlights the main biomarkers analyzed, including inflammatory cytokines, neurotrophic factors, mitochondrial proteins, and glial markers. Furthermore, the microRNAs identified as differentially expressed in response to tumor presence or chemotherapy are listed, along with the likely associated mechanisms, such as the regulation of neurogenesis, neuroinflammation, apoptosis, and synaptic plasticity.

As shown in Table 3, the selected studies identified a variety of neuropsychological alterations, with findings in domains such as memory, attention, executive function, and learning. These alterations are accompanied by identifiable changes in specific regions of the central nervous system (CNS), such as the hippocampus and prefrontal cortex, and are associated with mechanisms such as neuroinflammation, synaptic dysfunction, oxidative stress, blood–brain barrier (BBB) dysfunction, and apoptotic signaling.

Among the information presented in Table 2 and Table 3, doxorubicin was the most commonly investigated chemotherapy agent, appearing in four of the six selected studies (#2, #3, #5, and #6). Cyclophosphamide was cited in two of the seven studies, most often in combination with doxorubicin and paclitaxel as part of polychemotherapy regimens (#3 and #6). Cisplatin was cited in one study and was associated with mitochondrial dysfunction and hippocampal apoptosis (#4).

Regarding microRNAs, miR-155 was the most cited miRNA, present in two distinct studies (#1 and #2). The other miRNAs were cited in only one article each.

### 3.2. In Silico Analysis of miRNAs Involved in Neurological Changes Induced by Chemotherapeutic Agents

Based on the miRNAs found in the search, in addition to miR-155-5p, two miRNAs were selected based on pathway compatibility obtained in DIANA micro-CDS: miR-21-5p and miR-125b-5p. Analyses were performed with all miRNAs described in the six research articles, but combining them did not yield results directly related to the study. Therefore, it was decided to continue the study with only miR-155-5p, miR-21-5p, and miR-125b-5p. All selected miRNAs were verified in miRBase to confirm their mature sequence and conservation across species. Since five of the six studies used rats as the sample population, the species *Rattus norvegicus* was included in the search.

While miR-155-5p has no experimental validation, miR-21-5p and miR-125b-5p have already been experimentally cloned [28].

A target prediction analysis of the selected miRNAs was then performed using bioinformatics tools. Applying the pre-determined cutoffs in the methodological design, 245 target genes were found for miR-155-5p: 11 from TargetScan, 82 from miRDB, and 152 from DIANA. For miR-21-5p, 149 genes were listed in total, with 17 results from TargetScan, 66 from miRDB, and 66 from DIANA. Finally, for miR-125b-5p, 746 target genes were found, with 380 results from TargetScan, 143 from miRDB, and 223 from DIANA. The list of all target genes can be found in Appendix A. Table A1 lists the main target genes found for each miRNA in their respective databases, considering only the genes intersected between the databases.

Among the three miRNAs studied, considering the database used, it was not possible to identify common target genes predicted for the analyzed species (*Rattus norvegicus*). However, miR-155-5p and miR-125b-5p presented the ETS1 gene as a common target.

Due to the limited number of studies performed with miRNAs from the studied species, for inference purposes, the target genes found were checked for the existence of orthologs in the human genome. Of the target genes predicted by miR-155-5p, only three genes do not have human orthologs (RGD1563159, LOC691277, RGD1560028). For miR-125b-5p, only the NECAB3 gene does not have a human orthology, while for miR-21-5p, all target genes have orthologs.

Therefore, although the experimental models in the studies discussed in this review were based on mouse studies, we used the human miRNA reference for pathway analysis. This decision was based on two key considerations: (1) the vast majority of pathway and genetic interaction data in DIANA are reserved for the human genome, which offers better functional resolution and biological interpretation, and (2) the cross-species comparison revealed that almost all predicted targets of the selected miRNAs, approximately 99.6%, have confirmed human orthologs. Given the high degree of sequence conservation of miRNAs and their regulatory targets across animals, we considered this minimal divergence acceptable for extrapolating biologically relevant signaling pathways.

**Figure 2:**
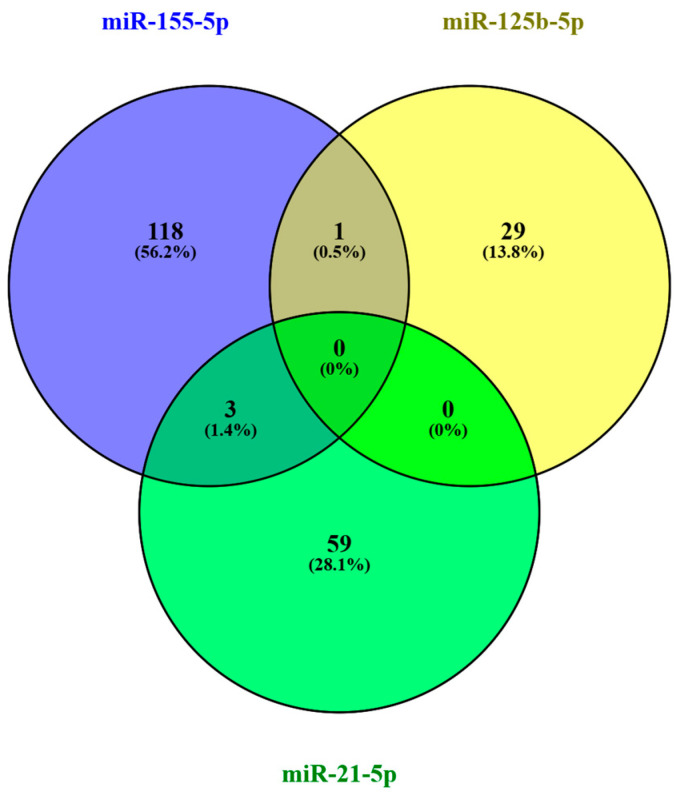
Experimentally validated target-genes of miR-21-5p, miR-125b-5p, miR-155-5p.

From the analysis performed on the DIANA TarBase v9 platform, which evaluated human target genes of miR-21-5p, miR-125b-5p, and miR-155-5p, 210 experimentally validated target genes were identified for the selected miRNAs, considering only evidence of direct interactions confirmed by luciferase assays, Western blot, and qRT-PCR. Among the identified targets, 122 genes were associated with miR-155-5p, 62 with miR-21-5p, and 31 with miR-125b-5p, indicating that miR-155-5p has the highest number of direct experimental validations among the miRNAs evaluated. Figure 2 shows the comparison between the target genes of each miRNA, indicating that three genes (APAF1, DOCK1, PDCD4) are common targets between miR-155-5p and miR-21-5p, and one gene (TP53INP1) is a common target between miR-155-5p and miR-125b-5p. APAF1, DOCK1, PDCD4, and TP53INP1 are genes associated with the regulation of apoptosis, cell migration, and tumor suppression, frequently modulated by miRNAs in pathological processes such as cancer. In this analysis, no common target gene was identified among the three miRNAs.

**Figure 3:**
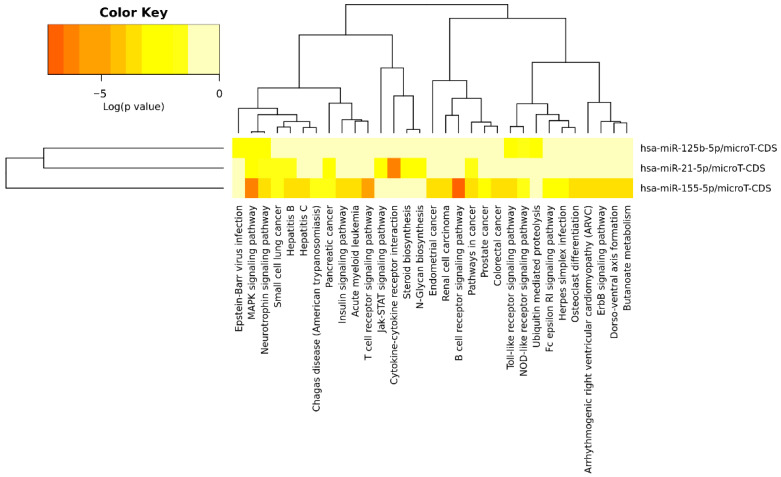
Heatmap showing the cell signaling pathways related to the three miRNAs in this study, according to microT-CDS.

**Figure 4:**
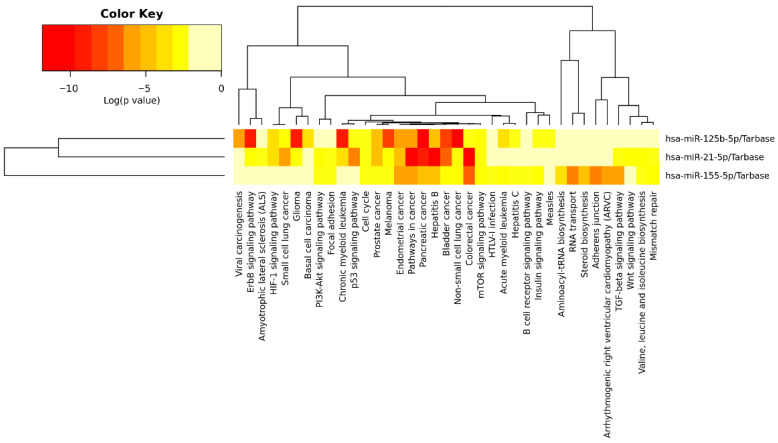
Heatmap showing the cell signaling pathways related to the three miRNAs in this study, according to TarBase.

Figure 3 shows the signaling pathways associated with the three miRNAs according to the microT-CDS database, while Table 4 presents these pathways, the *p*-value, the number of involved genes, and the corresponding miRNAs. Figure 4 displays the pathways identified through TarBase, and Table 5 provides a detailed list of these pathways.

Among the KEGG pathways demonstrated in the analysis, the cancer pathways, p53, mTOR, cell cycle, PI3K-Akt, Wnt, TGF beta, focal adhesion, neurotrophins, cytokine-cytokine receptor interaction, adhesion junction, Toll-like receptor, NOD-like receptor, and ErbB stand out. KEGG pathways represent functional maps that integrate genes and proteins within biological processes and signaling networks, allowing the identification of shared molecular mechanisms regulated by the selected miRNAs. These pathways were highlighted due to their significance in the present study and because they involve more than one miRNA, enabling protein-protein and protein-miRNA interaction analyses. It can be observed that several pathways related to specific cancer types demonstrated a relationship with miR-21-5p, miR-125b-5p, and miR-155-5p, but are not directly linked to neurological alterations. Considering all the signaling pathways highlighted, 180 genes were involved, which could be modulated directly or indirectly by the three miRNAs studied.

Comparing the 210 target genes identified as experimentally validated and the 180 genes involved in the highlighted signaling pathways, six genes in common can be identified: ERBB3, E2F3, RPS6KA1, SMO, and TP53. These genes are involved in critical pathways of proliferation, cell differentiation, and stress response, acting in processes such as cell cycle progression, growth signaling, and apoptosis control, and are frequently dysregulated in several types of cancer.

**Figure 5:**
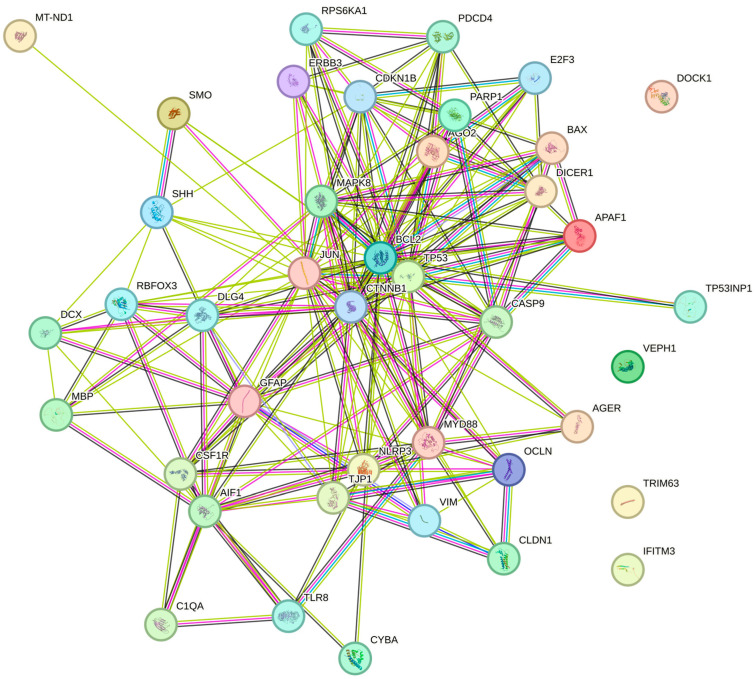
Protein–protein interaction (PPI) analysis using STRING v.11.0 software.

Considering the various analyses of possible target genes, 42 genes were selected for protein–protein interaction (PPI) analysis using the STRING v.11.0 software, with the result of the interaction shown in Figure 5.

Based on the data presented by the PPI network analysis, a total of 41 nodes (proteins) and 196 interactions (edges) were observed, with an average degree of connection of 9.56 and an average local clustering coefficient of 0.665. The number of interactions observed is significantly higher than expected for a random set of proteins with the same characteristics (expected interactions: 70), as evidenced by a *p*-value for PPI enrichment below 1.0 × 10^−16^. These results indicate that the analyzed proteins are functionally connected to each other to a degree much greater than chance, suggesting that they act in a coordinated manner in common biological pathways or related cellular processes.

The genes/proteins in the network are strongly associated with biological processes related to glial cell development and activation, including gliogenesis, astrocyte differentiation, and development, as well as glial cell differentiation, activation, and development. Furthermore, relevant immunological and inflammatory processes were identified, such as leukocyte activation during the inflammatory response, as well as pathways associated with the regulation of neuronal apoptosis and neuronal death. These results indicate that the analyzed proteins act in a coordinated manner in the regulation of neuroinflammatory and neurodegenerative events, in addition to playing central roles in glial development and maintenance, suggesting possible involvement in pathological conditions such as central nervous system injuries and neuroinflammatory diseases.

Furthermore, three main functional clusters were identified, notably Cluster 1, associated with colorectal cancer and apoptosis; Cluster 2, with diverse processes such as transverse myelitis and distal dendrites; and Cluster 3, related to the upregulation of interleukin-1 beta production. Analysis of the three clusters revealed the involvement of genes in distinct and relevant biological processes: Cluster 1 is primarily associated with neuronal apoptosis and response to genotoxic damage, such as gamma and UV radiation; Cluster 2 groups genes related to central nervous system development, including gliogenesis and astrocyte maturation; and Cluster 3 focuses on the regulation of inflammatory cytokine production, such as interleukin-1 beta. These findings suggest the coordinated role of genes in cellular responses to stress, inflammation, and glial differentiation.

## 4. Discussion

The articles analyzed in this study demonstrate that both the presence of extracranial tumors and the administration of chemotherapy drugs can trigger significant cognitive changes through neuroinflammatory, epigenetic, and metabolic mechanisms. This reinforces the idea that the chemobrain phenomenon is not exclusively a direct consequence of pharmacological toxicity but also results from the complex relationship between the systemic tumor microenvironment and the nervous system—a phenomenon also described as tumor brain (#3) [29].

### 4.1. Chemotherapy Agents and Their Neurocognitive Mechanisms

Among the chemotherapeutic agents investigated, doxorubicin, cyclophosphamide, paclitaxel, cisplatin, methotrexate, topotecan, and crizotinib were associated with deficits in memory, attention, learning, and executive functions, as well as neurobiological alterations such as reduced brain-derived neurotrophic factor (BDNF), mitochondrial dysfunction, loss of blood–brain barrier (BBB) integrity, and glial activation (#1, #2, #3, and #4). Similar alterations were observed in tumor-bearing animals without chemotherapy exposure, indicating an autonomous role of the tumor in modulating the brain microenvironment [29,30].

Retrospective cohort studies on methotrexate-induced cognitive impairment show predominant features of encephalopathy and polyradiculopathy, as well as clinical manifestations such as seizures and altered mental status. Other findings, more pronounced in oncological contexts such as leukemia, include neuroimaging abnormalities in the subcortical and periventricular white matter of the frontal lobes, as well as in the cerebellar peduncles and brainstem. Most of these events are mild and reversible upon discontinuation of the drug [31,32].

Conversely, in the context of autoimmune diseases, the anti-inflammatory activity of methotrexate may contribute to reducing cognitive damage caused by conditions such as rheumatoid arthritis. Long-term methotrexate use is associated with improvement in cognitive dysfunction, particularly regarding memory and attention, and with a reduced risk of developing dementias [33,34,35]. Other rheumatologic conditions, such as Sjögren’s syndrome, also induce significant cognitive impairment; however, methotrexate use contributes little to its reversal [36,37].

In animal models, methotrexate has been shown to cause spatial memory deficits, in addition to histopathological changes such as reduced hippocampal cell proliferation, decreased numbers of oligodendrocytes, and structural alterations in the corpus callosum. Furthermore, methotrexate is hypothesized to contribute to blood–brain barrier dysfunction by inducing senescence in endothelial and microglial cells [37,38,39].

Furthermore, some studies have already pointed to doxorubicin as responsible for the increase in neuroinflammation, evidenced by the upregulation of pro-inflammatory mediators, markers of oxidative stress and proteins related to apoptosis in the hippocampus and other brain regions, including the reduction in BDNF, which is fundamental for neurogenesis and synaptic integrity [40,41,42]. Agents such as cisplatin damage neuronal DNA and increase the production of reactive oxygen species. Paclitaxel induces neuroinflammation and neuronal necroptosis via RIP3 activation, and 5-fluorouracil alters hippocampal dendritic morphology [43].

### 4.2. microRNA-Related Molecular Pathways and Biological Mechanisms

At the molecular level, microRNAs stand out as central epigenetic mediators of these alterations. Pro-inflammatory miRNAs, such as miR-155, have been identified as intensifying cognitive impairment by promoting BBB disruption and neuroinflammation (#1), as well as neuroprotective miRNAs, such as miR-429-3p, miR-124, and miR-132, associated with the regulation of neurogenesis, synaptic function, and cellular homeostasis (#2 and #4). This corroborates other studies on miR-155, which show the molecule as a pro-inflammatory mediator, modulator of microglia and astrocytic activation, and its involvement in the dysregulation of blood–brain barrier function via the downregulation of adhesion proteins [44,45,46].

Furthermore, El-Derany et al. (2021) [23] observed that some miRNAs, including miR-155, were involved in the recovery of structural and cognitive deficits via exosomal delivery of mesenchymal stem cells, which modulate the Wnt/β-catenin and Hedgehog pathways, both functionally aligned with the neurotrophin and MAPK axes identified in silico.

Other experimental studies have also demonstrated that miR-155 upregulation in brain endothelial tissue mimics cytokine-induced dysregulation of the BBB, while its inhibition preserves its integrity and reduces infiltration during pro-inflammatory states [44]. In experimental models of Alzheimer’s disease, miR-155 modulates microglial phenotypes, with evidence suggesting that its deletion may promote a protective microglial state, reduce synaptic degradation, and improve cognitive outcomes [47].

The role of miR-155-5p in neuroinflammation can also be corroborated by the results of computational analysis. Chen et al. (2025) [22] demonstrated that miR-155 inhibition preserved BBB integrity and reduced the expression of inflammatory cytokines (IL-6, TNF-alpha). Similarly, DIANA miRPath analysis revealed significant enrichment in cytokine-cytokine receptor interaction pathways, Toll-like receptors, and NOD-like receptors, known to regulate microglial activation and neuroimmunological crosstalk. (#1; #4) [46]. On the other hand, these pathways were also highlighted in downregulation processes of pro-inflammatory miRNAs, such as miR-146a and miR-34a, which could guarantee neuroprotection (#4) [48].

Some microRNAs, such as miR-21 and miR-324, have been linked to increased axonogenic processes, suggesting the presence of oncologically orchestrated signaling for different cells through the packaging of specific exosomes and release of microRNAs [49]. Abnormal hypermethylation of miRNA genes is commonly observed during tumor development and results in the downregulation of tumor suppressor miRNAs [50]. Kovalchuk and collaborators (2017) also observed that miRNAs, such as the let-7 family, miR-429-3p and miR-409-3p, which act as tumor suppressors or neuroprotectors, can also have negative effects in inflammatory and neurotoxic contexts.

Also noteworthy is the downregulation of BDNF, observed in progesterone-positive breast cancer (PR + BC) models, associated with the overexpression of miRNAs miR-191-5p, the miR-183/96/182 cluster, and the miR-200 family, which have direct or indirect targets in BDNF (#3). Consistent with these findings, a study indicated a complex regulatory relationship between BDNF and microRNAs in nervous tissue. While BDNF stimulates miRNA expression, these molecules generally inhibit BDNF expression, forming a negative feedback loop. This balance is dysregulated in neurodegenerative diseases such as Alzheimer’s, where a decrease in BDNF levels is also observed [51]. Thus, the drop in BDNF levels, which represents a fundamental element for synaptic plasticity, learning and memory, may demonstrate an important link between molecular alterations and cognitive symptoms in cancer patients.

The neurotrophin signaling pathway, enriched in miR-155 targets in KEGG data, is important for synaptic repair and neurogenesis. Furthermore, Taha et al. (2023) [52] highlighted the dysregulation of BDNF/CREB signaling, which may be modulated by miRNAs to restore neuroplasticity in methotrexate-treated rats. Thus, the in silico prediction of BDNF regulatory pathways aligns with molecular data from several studies, confirming that the modulation of miRNAs targeting MAPK, neurotrophin, and Wnt pathways may hold translational potential.

The NF-κB signaling pathway was highlighted by Nasr et al. (2024) [26] as an important mediator of cognitive impairment. In this study, doxorubicin-induced miR-34a upregulation suppressed SIRT-1, leading to NLRP3 activation and increased neuroinflammation and apoptosis. Similarly, a 2024 study cites the relationship between miR-124 and miR-129 downregulation and NF-κB pathway activation in postoperative neurocognitive pathologies, while miR-190a-3p supplementation reduced oxidative stress and NF-κB activation [48]. This suggests that some signaling pathways may play a fundamental role in neuroinflammation and cognitive dysfunction in cancer patients.

The results of the microT-CDS and TarBase analyses demonstrated the importance of the PI3K-Akt, mTOR, and TGF-β pathways for the three miRNAs studied. These signaling cascades govern cell survival, mitochondrial function, and synaptic integrity. Experimental results from 2020 reinforce that the downregulation of miR-429-3p during cisplatin exposure impaired mitochondrial regulation, increasing caspase-9 and decreasing mt-ND1, which was reversed with dexmedetomidine (#4), suggesting a functional intersection with mTOR pathways and cell cycle regulation observed in the bioinformatics data.

Furthermore, the overexpression of Ago2, a protein important in the processing and action of microRNAs, identified in the prefrontal cortex of PR + BC animals, suggests that modulation of the miRNA machinery may also play a regulatory role in the intensity of neurological damage (#3). Other studies have observed that the Ago2 protein is essential for miRNA homeostasis and cellular development, especially in glutamatergic neurons, affecting the formation of the neurovascular unit, neuronal positioning, and the formation of the blood–brain barrier. The stability of this protein is dependent on the abundance of miRNAs, and its dysregulation may be associated with neuroinflammation [53].

The clinical implications of these results may be essential for a shift in therapeutic paradigms. Identifying circulating or tissue miRNAs as biomarkers of cognitive susceptibility may aid in the early screening of patients at risk for cognitive impairment, while treatment strategies based on exogenous miRNAs, miRNA antagonists, or extracellular vesicles may represent potential future approaches. The administration of mesenchymal stem cell-derived exosomes containing neuroprotective miRNAs, such as miR-21, miR-125b, and miR-132, showed significant restorative effects on cognitive function in a chemobrain animal model (#2). This is also demonstrated by a 2019 study in which an animal model of Alzheimer’s disease achieved significant cognitive improvement by stimulating neurogenesis in the subventricular zone after administration of mesenchymal stem cell-derived exosomes [54]. Other studies have also mentioned miR-21 and its role in neuroprotection and promotion of neuroplasticity, with miR-17-92 and miR-124 also being mentioned [55].

Nasr et al. (2024) [26] further describe the role of oxidative stress in chemotherapy-induced cognitive dysfunction, citing increased reactive oxygen species and malondialdehyde, as well as reduced glutathione, which were reversed by empagliflozin. Something similar was observed in a 2023 study involving methotrexate, where BDNF expression decreased and oxidative stress increased, which was reversed with dexmedetomidine, demonstrating a possible shared protective mechanism via antioxidant modulation [52]. Additionally, there are discussions about shared pathways between Alzheimer’s disease and cancer, involving some mitochondrial abnormalities and oxidative stress [56].

The in silico analysis corroborates and deepens the experimental findings of the selected original studies by identifying that miR-21-5p, miR-125b-5p, and miR-155-5p have targets in highly convergent signaling pathways that regulate processes highlighted in all the studies analyzed: neuroinflammation, oxidative stress, neuronal apoptosis, and synaptic plasticity. The common findings between the computational predictions and the in vivo results of the articles attest to the translational value of these miRNAs as potential biomarkers or therapeutic targets in the chemobrain context.

However, it should be recognized that the heterogeneity of animal models, the varied tumor types and therapeutic regimens, and the different study approaches hinder a more generalized interpretation of the results. The scarcity of behavioral studies integrated with molecular analysis of miRNAs may represent a limitation to an assertive understanding of the topic and the direct application of the findings to a clinical context.

## 5. Conclusions

This study aimed to identify the main microRNAs associated with chemotherapy-induced neuropsychological changes, as well as map their interaction networks and the biological pathways involved. Through an integrative literature review and bioinformatics analyses, we gathered relevant evidence on the central role of miRNAs in mediating neuroinflammatory, apoptotic, and oxidative stress processes in the chemobrain context.

Considering the genetic transposition between *Rattus norvegicus* and the human species, the data obtained demonstrate that miRNAs such as miR-155-5p, miR-21-5p, and miR-125b-5p have targets in highly conserved signaling pathways functionally linked to neuropsychological alterations observed in animal models, such as the MAPK, PI3K-Akt, mTOR, neurotrophin, and cytokine receptor pathways. Furthermore, in silico analysis revealed the translational potential of these miRNAs, both as biomarkers of cognitive susceptibility and as promising therapeutic targets. However, neuropsychological changes are not limited to cognitive impairment, which reinforces the need for more studies addressing other neuropsychological domains, as well as studies with species other than murine models.

The findings suggest that chemobrain is a multifactorial phenomenon, not limited to pharmacological toxicity, but also influenced by the presence of tumors and systemic modulation of the central nervous system. The changes observed in the reviewed studies, such as blood–brain barrier dysfunction, glial activation, and reduced BDNF, reinforce the complexity of the mechanisms involved.

Among the study’s limitations is the scarcity of integrated research combining behavioral assessment with molecular analysis of miRNAs, in addition to the heterogeneity of experimental models and chemotherapeutic agents used. Therefore, future studies are suggested to explore in greater depth the specific effects of different therapeutic regimens on miRNA expression in humans, as well as the development of therapeutic interventions based on microRNA modulation or the administration of exosomes containing neuroprotective miRNAs.

Thus, this work contributes to the understanding of the molecular mechanisms involved in cognitive changes associated with cancer and its therapy, pointing out ways for the personalization of oncological treatment with a focus on preserving patients’ cognitive function.

## Figures and Tables

**Table 1:** Characteristics of the included studies.

Identification	Study	Study Design	Population Sample	Country of Origin	Objectives
**#1**	Chen et al., 2025 [22]	Experimental study	Rats	China	To explore the role of miR-155 in methotrexate-induced cognitive impairment, evaluating whether miR-155 inhibition can restore BBB integrity and improve cognitive performance in an animal model.
**#2**	El-Derany et al., 2021 [23]	Experimental study	Rats	Egypt	To evaluate the therapeutic potential of bone marrow mesenchymal stem cells and their exosomes in reversing doxorubicin-induced cognitive deficits in rats, focusing on the role of exosomal microRNAs in modulating neuroinflammation and neurogenesis.
**#3**	Kovalchuk et al., 2017 [24]	Experimental study	Mice	Canada, United States	To characterize the global impact of extracranial tumor growth and chemotherapy on the brain microRNAome in a mouse TumorGraft model, focusing on prefrontal cortex alterations and identifying miRNA traits associated with chemobrain.
**#4**	Li et al., 2020 [25]	Experimental study	Rats	China	To investigate the role of miR-429-3p in mitigating cisplatin-induced neurotoxicity and cognitive impairment in rats, especially via modulation of mitochondrial apoptotic pathways, and to evaluate the protective effect of dexmedetomidine.
**#5**	Nasr et al., 2024 [26]	Experimental study	Rats	Egypt	To investigate the neuroprotective effects of empagliflozin against doxorubicin-induced chemobrain in rats, focusing on the modulation of oxidative stress, inflammation, apoptosis and the role of miRNA-34a and lncRNA HOTAIR in these mechanisms.
**#6**	Zare et al., 2025 [27]	Experimental study	Rats	United States, Iran and Australia	To conduct a comparative transcriptomic analysis between cancer-related cognitive impairment and neurodegenerative diseases, identifying shared molecular traits and pathways potentially regulated by miRNAs.

**Table 2:** Main chemotherapeutic agents and microRNAs involved.

Study	Chemotherapeutic Agent	Analysis Technique Used	Main Molecular Markers	Main miRNAs	Mechanisms Related to the microRNAs
**Chen et al., 2025 (#1)** [22]	Methotrexate (MTX)	RT-qPCR of miR-155 in brain tissue	1. Decreased claudin-1, ZO-1, and occludin (BBB junctions); 2. Increased TNF-alpha, IL-6, and IL-1 beta; 3. Increased BBB permeability	miR-155 increases after MTX, reversed with anti-miR-155	It contributes to inflammation and loss of BBB integrity; its inhibition with anti-miR-155 improves spatial memory and restores BBB junctions.
**El-Derany et al., 2021 (#2)** [23]	Doxorubicin (DOX)	RT-qPCR of exosomal miRNAs	1. Decreased BDNF, DCX, PSD95, NeuN, MBP, beta-catenin, Shh; 2. Increased IL-6, TNF-alpha, BAX, GFAP, IBA-1	1. miR-21-5p, miR-124-3p, miR-125b-5p, miR-132-3p, miR-146a-5p, 2. miR-155-5p	1. Anti-inflammatory and neuroprotective via BMSC exosomes; regulate apoptosis, neurogenesis, synaptogenesis, and Wnt/beta-catenin and Hedgehog pathways. 2. Pro-inflammatory.
**Kovalchuk et al., 2017 (#3)** [24]	Doxorubicin, cyclophosphamide, paclitaxel (DCP), topotecan (TOP) e crizotinib (CRIZ)	NGS (Next-Generation Sequencing) of small RNAs	1. Decreased BDNF (mainly in PR + BC) 2. Increased Ago2 (miRNA processing) 3. No significant effect on Dicer.	1. miR-191-5p increased in all treated and untreated tumor groups, targeted by BDNF. 2. miR-100-5p increased in TNBC and TNBC/DCP. 3. miR-200 family (miR-200a/b/c, miR-141, miR-429) increased in PR + BC, PR + BC/TOP, and PR + BC/CRIZ. 4. miR-183/96/182 cluster increased in PR + BC, targeted by BDNF. 5. let-7 family (let-7a/b/c/g/i) increased in several groups, especially PR + BC. 6. miR-22-3p, miR-409-3p decreased in PR + BC.	1. Associated with neurological disorders and BDNF downregulation. 2. Involved in mitochondrial dysfunction and PI3K/Akt/mTOR; linked to Alzheimer’s disease. 3 and 4. Regulate neuronal differentiation and inflammation; associated with neurotoxicity and brain tumors/metastases. 5. Tumor suppressor, but may have negative effects in the neurological context (associated with senescence and inflammation). 6. Neuroprotective; its reduction may have deleterious effects.
**Li et al., 2020 (#4)** [25]	Cisplatin	RT-qPCR of miR-429-3p in hippocampal tissue	1. Decreased mt-ND1 (mitochondrial DNA). 2. Increased caspase-9 (apoptosis). 3. Mitochondrial degeneration and structural changes in hippocampal neurons.	miR-429-3p reduced after cisplatin and increased with dexmedetomidine	Regulates apoptosis and mitochondrial function, its overexpression decreases caspase-9 and increases mt-ND1, protecting against neuronal damage
**Nasr et al., 2024 (#5)** [26]	Doxorubicin	RT-qPCR	SIRT-1, MuRF-1, PARP-1, NLRP3, FOXO-1, JNK, NF- κB	miR-34a, lncRNA HOTAIR	It promotes neuroinflammation and apoptosis via suppression of SIRT-1 and activation of the NLRP3 pathway. This lncRNA is involved in the regulation of inflammatory and apoptotic signaling, both of which are downregulated by empagliflozin, reducing neuroinflammation and neuronal apoptosis.
**Zare et al., 2025 (#6)** [27]	Doxorubicin, cyclophosphamide, paclitaxel, mithramycin	RNA-Seq (complete transcriptome)	C1qa, TLR8, IFITM3, GFAP, CSF1R, CDKN1B, VIM, JUN, CYBA	miR-509-3p, miR-3	Involved in neuroinflammation, synaptic dysfunction and cell cycle, in mechanisms common to CRCI and neurodegenerative diseases

**Table 3:** Main neuropsychological changes identified.

Study	Cognitive Domain	Identified Changes	Affected CNS Regions	Associated Mechanisms
**Chen et al., 2025 (#1)** [22]	Spatial memory, analyzed by the Morris water maze test.	Impairment in knowledge acquisition and memory performance, associated with increased permeability of the cerebral cortex and decreased expression of tight junction proteins (claudin-1)	Cortex and hippocampus, where changes in permeability and cytokine expression (TNF-alpha, IL-6, IL-1beta) occur.	Disruption of blood–brain barrier integrity, mediated by changes in tight junction proteins and inflammatory cytokines, with miR-155 having a protective role in maintaining BBB integrity and modulating neuroinflammation.
**El-Derany et al., 2021 (#2)** [23]	Spatial memory, short-term memory, contextual learning and avoidance memory, assessed by the Morris water maze, Y-maze and step-through avoidance.	Behavioral and cognitive deficits, hippocampal neurodegeneration, demyelination, increased apoptosis and inflammation, decreased neurotrophic and synaptic factors	Hippocampus and corpus callosum (associated with demyelination)	Oxidative stress, neuroinflammation (increased IL-6, TNF-alpha), activation of astrocytes and microglia (increased GFAP, IBA-1), apoptosis (increased BAX/Bcl2), inhibition of Wnt/Beta-catenin and Hedgehog pathways, with reversal mediated by exosomal miRNAs (miR-21-5p, miR-125b-5p).
**Kovalchuk et al., 2017 (#3)** [24]	Memory, processing speed, executive function, learning. There was no information about the tests conducted to study cognitive function.	Dysfunctions associated with tumor and chemotherapy	Prefrontal cortex	MicroRNA dysregulation, BDNF reduction
**Li et al., 2020 (#4)** [25]	Spatial memory and learning (Morris wáter maze test was used to study cognitive function).	Mitochondrial and hippocampal damage, involving neuronal apoptosis and mitochondrial dysfunction	Hippocampus	Cisplatin-induced downregulation of protective miRNAs (miR 429-3p), interaction with mitochondrial DNA (mt-ND1), activation of apoptotic pathways (via caspase-9) and subsequent neurodegeneration.
**Nasr et al., 2024 (#5)** [26]	Memory (Morris water maze test); motor function (rotarod test); and anxiety (open field test).	Impaired spatial learning and memory, reduced motor coordination, increased anxiety	Hippocampus (CA3, dentate gyrus), cerebral cortex	Doxorubicin-induced oxidative stress, inflammation, and apoptosis via the JNK/PARP-1/NLRP3 axis are modulated by empagliflozin through upregulation of SIRT-1 and downregulation of miR-34a and lncRNA HOTAIR, reducing neuronal damage and improving cognitive functions.
**Zare et al., 2025 (#6)** [27]	Memory, attention, language, learning, visual–spatial skills, motor and executive function. There was no information about the tests conducted to study cognitive function.	Gene patterns similar to Alzheimer’s disease and Parkinson’s disease; changes in neural activity; inflammation, cell cycle, and synapses	Hippocampus, prefrontal cortex, substantia nigra and other regions dependent on the animal model used	Neuroinflammation (C1qa pathway, TLR8, IFITM3), oxidative stress, cell cycle alterations, synaptic dysfunction, pathways shared with AD/PD (e.g., MYD88, AGE-RAGE, TGF-beta, PI3K-AKT-mTOR).

**Table 4:** Cell signaling pathways related to the three miRNAs in this study, according to results from the microT-CDS tool. Pathways highlighted in blue indicate those most closely related to chemotherapy-induced cognitive impairment.

	KEGG Pathways	*p*-Value	Genes (n)	miRNAs (n)	miRNAs Parte
**1**	**MAPK pathway**	**4.56 × 10^−10^**	**42**	**3**	**miR-21-5p, miR-125b-5p, miR-155-5p**
**2**	**Neurotrophin signaling pathway**	**1.70 × 10^−6^**	**22**	**3**	**miR-155-5p**
3	B cell receptor signaling pathway	3.01 × 10^−6^	10	1	miR-155-5p
4	T cell receptor signaling pathway	3.25 × 10^−5^	11	1	miR-21-5p
**5**	**Cytokine-cytokine receptor interaction**	**4.75 × 10^−5^**	**9**	**1**	**miR-21-5p, miR-125b-5p, miR-155-5p**
6	Hepatitis B	4.75 × 10^−5^	15	2	miR-21-5p, miR-155-5p
**7**	**Toll-like receptor pathways**	**9.40 × 10^−5^**	**16**	**2**	**miR-125b-5p, miR-155-5p**
8	Hepatitis C	0.000311	10	1	miR-155-5p
**9**	**Cancer pathways**	**0.000495**	**29**	**2**	**miR-21-5p, miR-155-5p**
10	Small cell lung cancer	0.001279	10	2	miR-21-5p, miR-155-5p
11	Acute myeloid leukemia	0.001438	6	1	miR-155-5p
12	Pancreatic cancer	0.001739	9	2	miR-21-5p, miR-155-5p
13	Renal cell carcinoma	0.001884	8	1	miR-155-5p
14	Arrhythmogenic right ventricular cardiomyopathy	0.002715	5	1	miR-155-5p
15	Epstein–Barr virus infection	0.002872	13	1	miR-125b-5p
16	Insulin signaling pathway	0.003294	10	1	miR-155-5p
17	Osteoclasts differentiation	0.004561	9	1	miR-155-5p
18	Endometrial cancer	0.004749	6	1	miR-155-5p
19	Colorectal cancer	0.006383	6	1	miR-155-5p
20	Ubiquitin-mediated proteolysis	0.007381	10	1	miR-125b-5p
21	Formation of the dorsoventral axis	0.008353	4	1	miR-155-5p
22	Herpes simplex infection	0.008683	11	1	miR-155-5p
23	Steroid biosynthesis	0.009789	2	1	miR-21-5p
**24**	**NOD-like receptors pathway**	**0.011221**	**9**	**2**	**miR-125b-5p, miR-155-5p**
25	Prostate cancer	0.011872	7	1	miR-155-5p
26	Fc epsilon RI signaling pathway	0.014263	6	1	miR-155-5p
27	ErbB signaling pathway	0.018125	8	1	miR-155-5p
28	Jak-STAT signaling pathway	0.021110	7	1	miR-21-5p
29	Chagas disease (American trypanosomiasis)	0.021710	6	1	miR-155-5p
30	Butanoate metabolism	0.026185	4	1	miR-155-5p
31	N-glycan synthesis	0.045596	2	1	miR-21-5p

**Table 5:** Cell signaling pathways related to the three miRNAs in this study, according to results from the TarBase tool. Pathways highlighted in blue indicate those most closely related to chemotherapy-induced cognitive impairment.

	KEGG Pathways	*p*-Value	Genes (n)	miRNAs (n)	miRNAs
1	Hepatitis B	<1 × 10^−16^	32	3	miR-21-5p, miR-125b-5p, miR-155-5p
**2**	**Cancer pathways**	**<1 × 10^−16^**	**58**	**3**	**miR-21-5p, miR-125b-5p, miR-155-5p**
3	Colorectal cancer	<1 × 10^−16^	23	3	miR-21-5p, miR-125b-5p, miR-155-5p
4	Pancreatic cancer	<1 × 10^−16^	24	3	miR-21-5p, miR-125b-5p, miR-155-5p
5	Bladder cancer	6.66 × 10^−16^	14	3	miR-21-5p, miR-125b-5p, miR-155-5p
6	Endometrial cancer	3.04 × 10^−13^	17	3	miR-21-5p, miR-125b-5p, miR-155-5p
7	Non-small cell lung cancer	6.26 × 10^−13^	15	3	miR-21-5p, miR-125b-5p, miR-155-5p
8	Chronic myeloid leukemia	3.62 × 10^−12^	13	2	miR-21-5p, miR-125b-5p
9	Melanoma	2.96 × 10^−11^	16	3	miR-21-5p, miR-125b-5p, miR-155-5p
10	Glioma	5.40 × 10^−10^	11	2	miR-21-5p, miR-125b-5p
**11**	**ErbB signaling pathway**	**1.07 × 10^−9^**	**9**	**2**	**miR-21-5p, miR-125b-5p**
**12**	**p53 signaling pathway**	**2.73 × 10^−8^**	**12**	**2**	**miR-21-5p, miR-125b-5p**
13	Prostate cancer	3.13 × 10^−8^	20	3	miR-21-5p, miR-125b-5p, miR-155-5p
14	Small cell lung cancer	1.77 × 10^−7^	13	2	miR-21-5p, miR-125b-5p
15	HIF-1 signaling pathway	3.64 × 10^−6^	12	2	miR-21-5p, miR-125b-5p
16	RNA transport	4.20 × 10^−6^	21	1	miR-155-5p
**17**	**Adherence junctions**	**7.68 × 10^−6^**	**12**	**1**	**miR-155-5p**
**18**	**TGF-beta signaling pathway**	**7.92 × 10^−6^**	**13**	**2**	**miR-21-5p, miR-155-5p**
19	Acute myeloid leukemia	1.97 × 10^−5^	9	2	miR-125b-5p, miR-155-5p
**20**	**mTOR signaling pathway**	**3.39 × 10^−5^**	**17**	**3**	**miR-21-5p, miR-125b-5p, miR-155-5p**
21	Arrhythmogenic right ventricular cardiomyopathy	8.36 × 10^−5^	9	1	miR-155-5p
22	Viral carcinogenesis	0.000302	5	1	miR-125b-5p
23	Steroid biosynthesis	0.000410	3	1	miR-155-5p
**24**	**Cell cycle**	**0.000650**	**22**	**3**	**miR-21-5p, miR-125b-5p, miR-155-5p**
25	Hepatitis C	0.001425	15	2	miR-125b-5p, miR-155-5p
26	Valine, leucine and isoleucine biosynthesis	0.001582	1	2	miR-21-5p, miR-155-5p
27	Basal cell carcinoma	0.001789	3	1	miR-125b-5p
28	Insulin signaling pathways	0.002669	17	2	miR-125b-5p, miR-155-5p
**29**	**PI3K-Akt signaling pathway**	**0.002841**	**39**	**2**	**miR-21-5p, miR-155-5p**
**30**	**Focal adhesion**	**0.005444**	**26**	**2**	**miR-21-5p, miR-155-5p**
31	HTLV-I infection	0.006208	22	1	miR-155-5p
32	Mismatch repair	0.006283	3	2	miR-21-5p, miR-155-5p
33	Aminoacyl-tRNA biosynthesis	0.010195	9	1	miR-155-5p
**34**	**Wnt signaling pathway**	**0.013940**	**10**	**1**	**miR-21-5p**
35	B cell receptors signaling pathway	0.021941	8	1	miR-155-5p
36	Chickenpox	0.025838	4	1	miR-125b-5p
37	Lateral amyotrophic sclerosis (LAS)	0.030180	5	1	miR-21-5p

## Data Availability

All data supporting the results of this study are openly available in the databases referenced in this manuscript.

## Appendix A

**Table A1:** Predicted target genes for miR-155-5p, miR-21-5p, and miR-125b-5p in the TargetScan, miRDB, and DIANA databases.

MiRNA	Databases			
	TargetScan + miRDB	miRDB + DIANA	TargetScan + DIANA	TargetScan + miRDB + DIANA
miR-155-5p	No target gene in common	ACTA1, ACTL7A, AICDA, ADNP2, BSDC1, CACUL1, CARHSP1, CSF1R, CSNK1G2, CRISP1, DHX40, DUSP14, DYNC1I1, EDNRB, EHD1, ETS1, FAM168A, G2E3, HBP1, HIVEP2, INPP5D, ITK, JADE1, LOC680039, LOC691277, MAP3K14, MRGPRX3, MRGBP, MYO1D, NFE2L2, NPY1R, OLFML3, PDE11A, PKN2, RC3H2, RCN2, RGD1307461, RGD1560028, RGD1563159, RPS6KA3, RREB1, SALL1, SEMA5A, SMARCA4, SPI1, TAB2, TENM3, TMEM165, TOMM20, TRIM32, UAP1, UPP2, VPS18, WBP1L, WFDC21, ZFP652	KDM5B, XKR4	TSHZ3, WEE1
miR-21-5p	No target gene in common	ARMCX1, BTG2, CASKIN1, CCL1, CCL20, EPHA4, FASLG, GANC, IL6R, JAG1, MRPL9, NFIB, PCSK6, PELI1, PLEKHA1, PPP1R3A, RAB11A, RASGRP1, RECK, SF3A1, SLC16A4, SPRY2, TIMP3, TMEM170A, VCL, ZFP367	AP1AR, MPRIP, YOD1	ARHGAP24, ELF2, KLF3, SMAD7, TGFBI
miR-125b-5p	AJUBA, CASP2, CCNJ, ENPEP, ESRRA, GGA2, HAPLN1, IER2, LIN28A, MAP3K11, MTF1, NCAN, NUP210, PCGF6, PI4K2B, RFXANK, SMURF1, SLC6A17, SYVN1, TMEM120B, TMEM135, TSPAN12, UBR7, VPS37B	ALG6, BAP1, CDC42BPG, CHFT8, CLEC4A1, CNTD1, CYP24A1, DYNLT3, EIF1AD, FAM83H, FLVCR2, FUT1, GLB1L2, KCNS3, LACTB, MAPK13, MKNK2, PHF20, PLB1, PTPN1, RABL6, SEC14L2, SPEG, THEM6, TMEM25, TMPRSS13, TRIAP1, VPS4B, WARS, ZFP281, ZFP518A, ZFP691	ABTB1, ANPEP, AREL1, ATXN3, CACNB1, CCNJL, CORO2A, CPSF6, DAAM1, DENND6A, DNAJC14, DPF2, EAF1, ENTPD4, ETS1, EVA1A, FAM118A, FAM131B, FAM134A, FOXN3, GCNT1, GOPC, IST1, KLC2, LRFN2, LRP4, LURAP1L, MAN1B1, MGAT4A, MXD4, MYO18A, MYT1, NIPAL4, NT5DC1, PPP2CA, PPAT, QSOX2, RAPGEF5, RAPGEFL1, RFX5, SH3BP5L, SIM1, SUV420H2, SZRD1, TAF9B, TDG, TMEM132E, TMEM136, VDR, WDR1, ZBTB7A	ABHD6, ACER2, ARID3B, BAK1, BMF, BLZF1, DRAM2, ESRRG, GALNT14, GALNT5, GRB10, GRSF1, GTPBP2, IL16, IRF4, ITGA8, KCNK10, MFSD9, NECAB3, NEU1, NPL, NRM, OLFML2A, OSBPL9, PAFAH1B1, PCTP, PLEKHA8, PRDM1, RAP1A, RBM20, RHOQ, SBNO1, SEL1L, SEMA4C, SEMA4D, SERTAD3, SLC25A35, SLC35A4, SLITRK6, SSTAT3, SSTR3, STARD13, STAT3, TAZ, TGOLN2, TLE3, TMEM168, TNFSF4, TOR2A, ZFYVE1
